# Protective Role of Nuclear Factor Erythroid-2-Related Factor 2 against Mechanical Trauma-Induced Apoptosis in a Vaginal Distension-Induced Stress Urinary Incontinence Mouse Model

**DOI:** 10.1155/2019/2039856

**Published:** 2019-03-06

**Authors:** Jianming Tang, Cheng Liu, Bingshu Li, Shasha Hong, Qiannan Li, Linlin Wang, Jie Min, Ming Hu, Yang Li, Songming He, Li Hong

**Affiliations:** Department of Gynecology and Obstetrics, Renmin Hospital of Wuhan University, Wuhan, Hubei Province 430060, China

## Abstract

Apoptosis and oxidative damage are involved in the pathogenesis and progression of stress urinary incontinence (SUI). Our previous results indicate that cell apoptosis and oxidative damage increase in a mouse model of mechanical injury-induced SUI and in fibroblasts treated with excessive mechanical strain. Nuclear factor erythroid-2-related factor 2 (Nrf2) is a well-characterized global antioxidant gene inducer that can reduce oxidative damage and apoptosis. Therefore, we predicted that Nrf2 may have a protective role in mechanical trauma-induced SUI. To test this hypothesis, a mouse model of vaginal distension- (VD-) induced SUI was established. Leak point pressure (LPP); levels of apoptosis, apoptosis-related proteins, and peroxidation products; and the activities of antioxidative proteins in the anterior vaginal wall were measured in wild-type (Nfe2l2^+/+^) C57BL/6 mice and *Nrf2*-knockout mice (Nfe2l2^−/−^). The results showed that *Nrf2* knockout aggravated VD-induced reduction in LPP, increase in cell apoptosis and peroxidation product levels, decrease in antioxidative protein activities, and alterations in apoptosis-related protein levels in the vaginal walls of mice. To further confirm the role of Nrf2 in mechanical trauma-induced apoptosis and SUI, VD was performed on mice overexpressing Nrf2 via *in vivo* transfection of LV-Nfe2l2. The results showed that Nrf2 overexpression significantly alleviated VD-induced abnormalities in the anterior vaginal wall. Taken together, our data suggested that Nrf2 is a potential protective factor in mechanical trauma-induced apoptosis in a mouse model of SUI. Antioxidative therapy may be a promising treatment for mechanical trauma-related SUI.

## 1. Introduction

Female pelvic floor dysfunction (PFD) is one of the most common benign gynecological diseases, which also include urinary incontinence (UI), pelvic organ prolapses (POP), and sexual dysfunction. Stress urinary incontinence (SUI) is one of the most common types of PFD in women. Epidemiological studies indicate that SUI has significant adverse effects on women's everyday life and causes great economic burden [1, 2]. Aging and vaginal delivery-induced pelvic injury are two of the most important risk factors of these disorders [3, 4]. The normal function of the female pelvic floor requires an intact anatomical structure, consisting of pelvic muscles, nerves, and connective tissues. The mechanical strength and toughness of the pelvic tissues are essential for maintaining the normal anatomical position and function of pelvic organs [5]. The vaginal wall plays an important role in maintaining the normal position and function of the vesical neck and urethra, which are attached to the anterior vaginal wall [5–7]. Vaginal delivery and chronic constipation or other causes of abdominal pressure increase the mechanical injury of pelvic connective tissues and muscular tissues. This can cause traumatic slackness and alterations of the normal anatomical position and function of pelvic organs, resulting in SUI and/or POP. Increased oxidative damage and apoptotic index have been reported in vaginal walls and pelvic connective tissues of PFD patients, especially in POP patients and patients with SUI induced by vaginal birth trauma or chronic abdominal pressure [8–11]. These data suggest that oxidative damage and apoptosis may contribute to PFD.

Mechanical stress can induce the accumulation of reactive oxygen species (ROS). Normal mechanical strain induces a physiological rise of ROS levels, while the excessive mechanical stress causes excessive ROS accumulation, eventually resulting in oxidative damage [12–16]. Oxidative damage has been reported to be involved in mechanical trauma-induced PFD [8, 9]. Nuclear factor erythroid-2-related factor 2 (Nrf2) is a well-characterized global antioxidant gene inducer. The nuclear translocation of Nrf2 can activate a series of antioxidant genes, such as glutathione peroxidase (*GPX*), superoxide dismutase (*SOD*), catalase (*CAT*), and heme oxygenase-1 (*HO-1*). Apoptosis is involved in the pathogenesis and pathological process of many types of diseases, including extracellular matrix (ECM) remodeling after tissue injury. Therefore, increased apoptosis may cause ECM remodeling and eventually result in the weakening of pelvic structures after various types of pelvic trauma, including pregnancy and vaginal delivery, pelvic surgery, or chronic abdominal pressure induced by chronic constipation. Our previous research indicates that mechanical injury increases ROS levels and induces oxidative damage and apoptosis in human uterosacral ligament fibroblasts (hUSLFs) and mouse connective tissue fibroblasts (L929) [8, 17]. In addition, the upregulation of antioxidative genes significantly alleviates mechanical trauma-induced cell apoptosis [8, 17]. Therefore, we predicted that Nrf2 may have a protective role in mechanical trauma-induced SUI, through the reduction of apoptosis.

In the present study, we explored the role of Nrf2 in mechanical trauma-induced cell apoptosis *in vivo*. The results showed that *Nrf2*-kncokout aggravated the vaginal distension- (VD-) induced reduction in leak pressure point (LPP), increase in cell apoptosis and peroxidation products, decrease in antioxidative protein activities, and alterations in apoptosis-related protein levels in vaginal walls of mice. On the contrary, *in vivo* overexpression of Nrf2 significantly alleviated VD-induced alterations in mice. Taken together, our data suggested that Nrf2 is a potential protective factor in mechanical trauma-induced apoptosis in a mouse model of SUI and in mechanical trauma-induced SUI. Antioxidative therapy may be a promising treatment for mechanical trauma-related SUI.

## 2. Materials and Methods

### 2.1. Mice and Study Design

Mouse breeding and experimentation were performed in compliance with the institutional guidelines and regulations of Wuhan University. All animal experiments in this study were approved by the Institutional Animal Care and Use Committee of Renmin Hospital of Wuhan University. We purchased wild-type (WT, Nfe2l2^+/+^) virgin female C57BL/6J mice from the Center for Animal Experiment of Wuhan University and *Nrf2*-knockout mice (KO, Nfe2l2^−/−^) from the Jackson Laboratory (Stock 017009; Bar Harbor, ME, USA). In the first part of this study, 60 WT virgin female C57BL/6J mice and 60 virgin female Nfe2l2^−/−^ mice were randomly divided into the following 4 groups: a nonoperated control (NC) group (WT-NC and KO-NC), a sham-operated group (WT-Sham and KO-Sham; catheter insertion and suture, but no water injected into the balloon), a VD7d group (WT-VD7d and KO-VD7d; underwent VD for 1 h, then LPP was measured on day 7 after VD), and a VD14d group (WT-VD14d and KO-VD14d; underwent VD for 1 h, then LPP was measured on day 14 after VD). There were 8 groups in total, with 15 mice per group. One day before LPP measurement, suprapubic tube implantations were performed. On the next day, LPP measurements were performed and then all mice were sacrificed and anterior vaginal tissues were harvested for the following experiments. In the second part of this study, 15 WT virgin female C57BL/6J mice were randomly divided into the following 3 groups, with 5 mice per group: a WT-VD7d group, as described above; a Vector-VD7d group, in which mice were injected with empty lentivirus vector (LV vector) before VD; and an OV-VD7d group, in which mice were injected with Nfe2l2-expressing lentivirus vector (LV-Nfe2l2) before VD. LPP measurements were then made and specimens were harvested on day 7 after VD. All mice used in this study were aged from 8 to 10 weeks. Mice were housed in the Animal Experimental Center and Institute of Model Animal of Wuhan University during these experiments. Details of the identification and validation of Nfe2l2^−/−^ mice are provided in Figure S1.

### 2.2. LV-Nfe2l2 Transfection *In Vivo*

LV-Nfe2l2, a recombinant lentiviral vector expressing Nrf2 (Nfe2l2, NM_010902) synthesized by Shanghai Genechem Co. Ltd. (Shanghai, China) was used for *in vivo* transfection to establish a Nrf2-overexpressing mouse model. *In vivo* transfection of LV-Nfe2l2 was performed as previously described [18, 19]. In short, a dose of 2 × 10^8^ of recombined lentivirus particles was dissolved in 300 *μ*L saline and delivered into mice using tail vein injection method. Mice were injected twice, with two days between each injection. Two weeks after injection, mice were treated with VD. Before *in vivo* transfection, the efficiency of overexpression was tested in L929 cells and LV-GFP was injected into the tail vein of mice to ensure *in vivo* transfection efficiency. Details of the validation of the *in vivo* transfection procedure are provided in Figure S2.

### 2.3. Vaginal Distention, Suprapubic Tube Implantation, and LPP Measurement

Vaginal distention, suprapubic tube implantation, and LPP measurement were performed as described in our previous study [16]. In brief, mice in the VD groups underwent vaginal distention with a modified 6 Fr Foley catheter filled with 0.3 ml of distilled water for 1 h. For the mice in sham-operated groups, only catheter insertion was administered and sutured for 1 h, but no water was injected into the balloon. One day before LPP measurement, an epidural catheter was implanted in the bladder. The LPP values were measured using a pressure transducer of a urinary dynamics detector (Nidoc970C, Weixin Medical of China). Details of vaginal distention, suprapubic tube implantation, and LPP measurement are provided in Supplementary Material S1.

### 2.4. TUNEL Assay for the Detection of Apoptosis

Cell apoptosis of the anterior vaginal walls of mice was detected using an ApopTag Plus Fluorescein In Situ Apoptosis Detection Kit (S7111; Chemicon International Inc., Temecula, California) according to the manufacturer's instructions. Then, slides were viewed and imaged with an Olympus BX51 fluorescence microscope (Olympus Corporation). The images were analyzed using Image-Pro Plus version 6.0 software (Media Cybernetics Inc., Rockville, MD, USA) and the mean percentage values of apoptosis-positive cells in the 200x microscopic field from every group were compared. Details of the TUNEL assay are provided in Supplementary Material S1.

### 2.5. Western Blot

Total proteins were extracted from mouse anterior vaginal wall tissues using RIPA buffer containing phenylmethanesulfonyl fluoride (PMSF). Western blot was performed as described in our previous research [16]. In brief, a BCA assay kit (Beyotime, China) was used to determine the protein concentrations according to the manufacturer's instructions. Then, protein loading buffer was added and denatured at 95°C for 10 min. After that, proteins were separated by 10% SDS-polyacrylamide gel electrophoresis (PAGE) and then transferred onto activated PVDF membranes. After blocking, the membranes were incubated with appropriate concentrations of primary antibodies at 4°C overnight, then followed by the incubation of fluorescence-labeled secondary antibodies (IRDye700 and IRDye800, goat anti-mouse/rabbit) for 1 hour at 37°C. Signals were detected with an Odyssey infrared imaging system (LI-COR Biosciences, Lincoln, NE, USA). Details of the information and dilutions of antibodies are provided in Supplementary Material S1.

### 2.6. MDA, CAT, GSH-PX, and T-SOD Measurement Assay

Total proteins were extracted from mouse anterior vaginal wall tissues using RIPA buffer containing PMSF. After protein concentration measurements with a BCA assay kit (Beyotime, China), MDA levels and activities of CAT, GSH-PX, and T-SOD were measured using MDA (S0131, Beyotime, China), CAT (S0051, Beyotime, China), GSH-PX (A005, Nanjing Jiancheng Bio-Engineering Institute Co. Ltd., China), and T-SOD (A001-1-1, Nanjing Jiancheng Bio-Engineering Institute Co. Ltd., China) measurement kits according to the manufacturers' instructions. Details of MDA, CAT, GSH-PX, and T-SOD measurements are provided in Supplementary Material S1.

### 2.7. Immunohistochemistry

All specimens of mouse anterior vaginal walls were embedded in paraffin, then cut into 4 *μ*m thick slices and fixed onto glass slides. The oxidative damage of anterior vaginal walls in mice was determined by immunohistochemical staining using an UltraSensitive SP kit 9710 (Fuzhou Maixin Biotech Co. Ltd., Fuzhou, China) according to the manufacturer's instructions as described in our previous study [20]. To evaluate the expression levels of 8-OHdG and 4-HNE, immunoactivity was quantified with an integrated optical density (IOD) value and the mean density was captured using Image-Pro Plus version 6.0 software (Media Cybernetics Inc., Rockville, MD, USA) using the following formula: mean density = IOD/area. Details of immunohistochemistry and the information and dilutions of antibodies are provided in Supplementary Material S1.

### 2.8. Statistical Analysis

All statistical analyses were performed with SPSS 21.0 (IBM Corporation, Armonk, NY, USA), and data are presented here as the mean ± SD. The data were further subjected to one-way analysis of variance. Differences between two groups were determined using the Dunnet *t*-test, and multiple means were compared by Tukey's test. *P* < 0.05 were considered statistically significant.

## 3. Results

### 3.1. *Nrf2* Knockout Aggravates Mechanical Trauma-Induced Reduction of LPP in Mice

**Figure 1:**
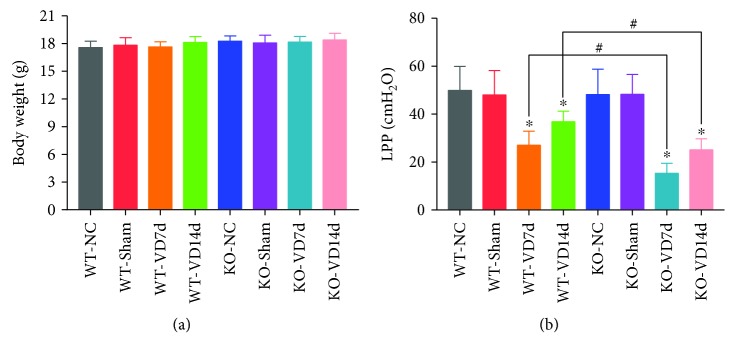
The body weights and LPP values of mice. (a) Body weights of mice in eight groups. (b) LPP values of mice in eight groups. ^∗^ *P* < 0.05 compared with the WT-VD group, ^#^ *P* < 0.05 between two groups. Every experiment was repeated 3 times. LPP: leak point pressure; VD: vaginal distension; WT-NC: nonoperated control group of wild-type mice; WT-Sham: sham-operated group of wild-type mice; WT-VD7d: VD group of wild-type mice and evaluated on day 7 after VD; WT-VD14d: VD group of wild-type mice and evaluated on day 14 after VD; KO-NC: nonoperated control group of *Nrf2*-knockout mice; KO-Sham: sham-operated group of *Nrf2*-knockout mice; KO-VD7d: VD group of *Nrf2*-knockout mice and evaluated on day 7 after VD; KO-VD14d: VD group of *Nrf2*-knockout mice and evaluated on day 14 after VD.

To determine whether Nrf2 is involved in the pathological process of mechanical trauma-induced SUI, LPP was measured. As shown in Figure 1(a), there was no difference in body weight among the eight groups of mice. The results (Figure 1(b)) also showed that there were no significant differences in LPP among the WT-NC, WT-Sham, KO-NC, and KO-Sham groups. The LPP of mice in the WT-VD7d group was significantly lower than the LPP of mice in the WT-NC, WT-Sham, KO-NC, and KO-Sham groups, but it was significantly higher than the LPP of mice in the KO-VD7d group. Besides, the LPP of mice in the WT-VD14d group was significantly higher than those in the WT-VD7d and KO-VD14d groups. There were no significant differences in LPP between the KO-VD7d and KO-VD14d groups. These results indicate that VD induced a reduction in LPP in mice, but a degree of recovery was seen on day 14 after VD. However, *Nrf2* knockout aggravated the mechanical trauma-induced reduction of LPP in mice and impaired their recovery capability.

### 3.2. *Nrf2* Knockout Exacerbates Mechanical Trauma-Induced Apoptosis in Anterior Vaginal Walls of Mice

**Figure 2:**
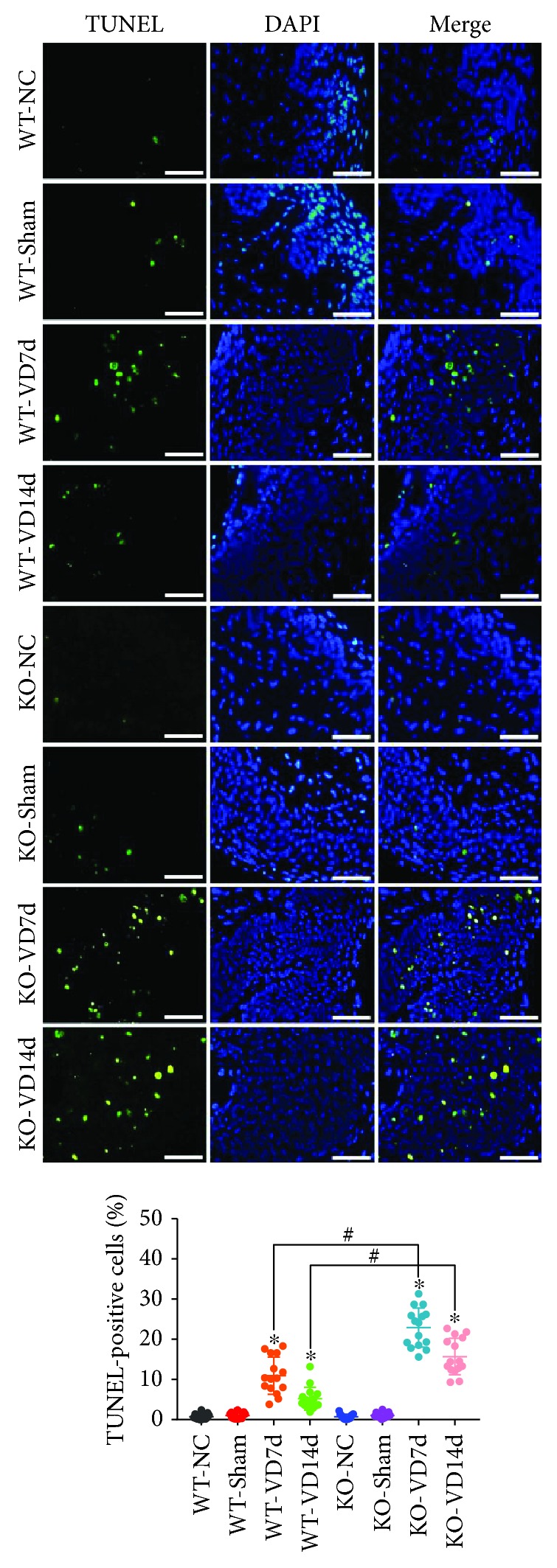
Apoptosis in the anterior vaginal wall of mice. TUNEL assay was used to detect apoptosis in the anterior wall of mice in eight groups. Green fluorescence is a positive signal for apoptosis. ^∗^ *P* < 0.05 compared with the WT-VD group, ^#^ *P* < 0.05 between two groups. Scale bars: 50 *μ*m. Every experiment was repeated 3 times and representative figures are presented here. VD: vaginal distension; WT-NC: nonoperated control group of wild-type mice; WT-Sham: sham-operated group of wild-type mice; WT-VD7d: VD group of wild-type mice and evaluated on day 7 after VD; WT-VD14d: VD group of wild-type mice and evaluated on day 14 after VD; KO-NC: nonoperated control group of *Nrf2*-knockout mice; KO-Sham: sham-operated group of *Nrf2*-knockout mice; KO-VD7d: VD group of *Nrf2*-knockout mice and evaluated on day 7 after VD; KO-VD14d: VD group of *Nrf2*-knockout mice and evaluated on day 14 after VD.

Apoptosis has been reported to be involved in the pathological process of PFD. Therefore, apoptosis was measured in the mouse anterior vaginal wall. As shown in Figure 2, apoptosis rates were very low in the WT-NC, WT-Sham, KO-NC, and KO-Sham groups, but they were significantly increased after VD. The apoptosis rate in the KO-VD7d group was significantly higher than that in the WT-VD7d group. In addition, the apoptosis rate in the WT-VD14d group was significantly decreased compared with that in the WT-VD7d group, but there was no difference between the KO-VD14d and KO-VD7d groups. These results suggest that VD-induced mechanical trauma significantly increased apoptosis in the anterior vaginal walls of mice, and this was aggravated by *Nrf2* knockout.

### 3.3. *Nrf2* Knockout Increases Mechanical Trauma-Induced Alterations in Apoptosis-Related Proteins in the Anterior Vaginal Walls of Mice

**Figure 3:**
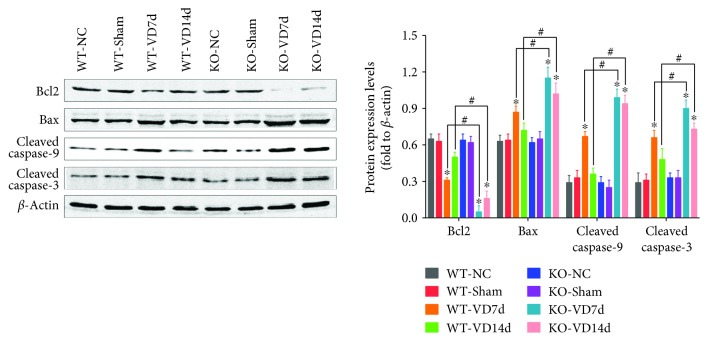
Levels of apoptosis-related proteins in the anterior wall of mice. Western blot was used to determine the levels of apoptosis-related proteins in the anterior wall of mice in eight groups. ^∗^ *P* < 0.05 compared with the WT-VD group, ^#^ *P* < 0.05 between two groups. Every experiment was repeated 3 times, and representative figures were presented here. VD: vaginal distension; WT-NC: nonoperated control group of wild-type mice; WT-Sham: sham-operated group of wild-type mice; WT-VD7d: VD group of wild-type mice and evaluated on day 7 after VD; WT-VD14d: VD group of wild-type mice and evaluated on day 14 after VD; KO-NC: nonoperated control group of *Nrf2*-knockout mice; KO-Sham: sham-operated group of *Nrf2*-knockout mice; KO-VD7d: VD group of *Nrf2*-knockout mice and evaluated on day 7 after VD; KO-VD14d: VD group of *Nrf2*-knockout mice and evaluated on day 14 after VD.

The protein levels of apoptosis-related proteins in the anterior vaginal walls of mice were measured by western blotting. As shown in Figure 3, there were no significant differences in the protein levels of Bcl2, Bax, cleaved caspase-3, or cleaved caspase-9 among the WT-NC, WT-Sham, KO-NC, and KO-Sham groups. However, levels of Bcl2 decreased and levels of Bax and cleaved caspases-3 and -9 increased after VD. Bcl2 protein levels in the KO-VD7d and KO-VD14d groups were significantly lower than those in the WT-VD7d and WT-VD14d groups, respectively. On the contrary, the protein levels of Bax and cleaved caspases-3 and -9 in the KO-VD7d and KO-VD14d groups were significantly higher than those in the WT-VD7d and WT-VD14d groups, respectively. These data indicate that VD activated apoptosis signaling via the downregulation of Bcl2, upregulation of Bax, and cleavage of caspases-3 and -9 in the anterior vaginal walls of mice. These data also show that *Nrf2* knockout promoted VD-induced activation of apoptosis signaling.

### 3.4. *Nrf2* Knockout Exacerbates Mechanical Trauma-Induced Oxidative Damage in the Anterior Vaginal Walls of Mice

**Figure 4:**
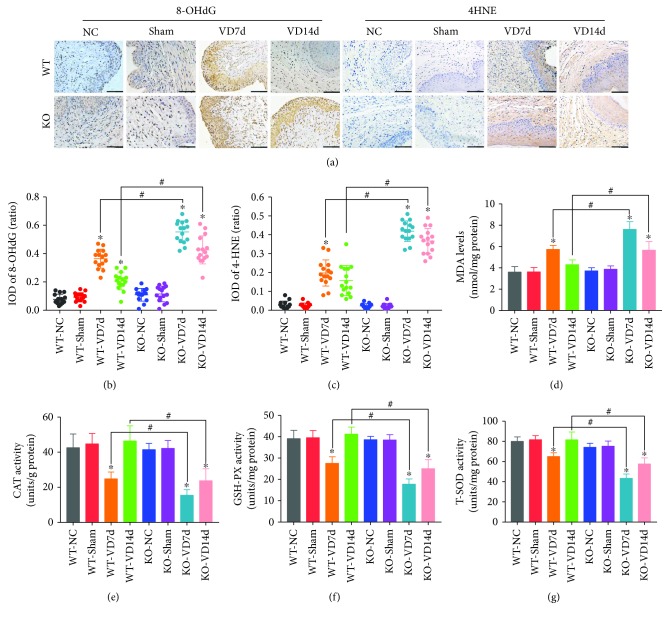
Oxidative damage and antioxidative capacity in the anterior wall of mice. (a–c) Immunohistochemistry was used to detect the levels of 8-OHdG and 4-HNE in the anterior wall of mice in eight groups. Brown represents the positive signal. Scale bars: 100 *μ*m. (d) The MDA measurement kit was used to detect the levels of MDA in the anterior wall of mice in eight groups. (e–g) CAT, GSH-PX, and T-SOD measurement kits were used to determine the activities of CAT, GSH-PX, and T-SOD, respectively, in the anterior wall of mice in eight groups. ^∗^ *P* < 0.05 compared with the WT-VD group, ^#^ *P* < 0.05 between two groups. Every experiment was repeated 3 times and representative figures are presented here. 8-OHdG: 8-oxo-2′-deoxyguanosine; 4-HNE: 4-hydroxynonenal; MDA: malondialdehyde; CAT: catalase; GSH-PX: glutathione peroxidase; T-SOD: total superoxide dismutase; VD: vaginal distension; WT-NC: nonoperated control group of wild-type mice; WT-Sham: sham-operated group of wild-type mice; WT-VD7d: VD group of wild-type mice and evaluated on day 7 after VD; WT-VD14d: VD group of wild-type mice and evaluated on day 14 after VD; KO-NC: nonoperated control group of *Nrf2*-knockout mice; KO-Sham: sham-operated group of *Nrf2*-knockout mice; KO-VD7d: VD group of *Nrf2*-knockout mice and evaluated on day 7 after VD; KO-VD14d: VD group of *Nrf2*-knockout mice and evaluated on day 14 after VD.

Our previous studies have shown that oxidative stress is involved in the pathological process of mechanical trauma-induced apoptosis and ECM remodeling in fibroblasts and in a mouse model of SUI [8, 16, 21]. Therefore, we measured the levels of peroxidation products and the activities of antioxidant proteins. As shown in Figure 4, there were no significant differences in the levels of 8-hydroxy-2′-deoxyguanosine (8-OHdG), 4-hydroxynonenal (4-HNE), or malondialdehyde (MDA) (Figures 4(a)–4(d)) or the activities of catalase (CAT), glutathione peroxidase (GSH-PX), or total superoxide dismutase (T-SOD) (Figures 4(e)–4(g)) in the anterior vaginal walls of mice among the WT-NC, WT-Sham, KO-NC, and KO-Sham groups. However, the levels of 8-OHdG, 4-HNE, and MDA in the WT-VD7d group were significantly higher than those in the WT-NC, WT-Sham, KO-NC, and KO-Sham groups, but they were significantly lower than those in the KO-VD7d group (Figures 4(a)–4(d)). On the contrary, the activities of CAT, GSH-PX, and T-SOD in the WT-VD7d group were significantly lower than those in the WT-NC, WT-Sham, KO-NC, and KO-Sham groups, but they were significantly higher than those in the KO-VD7d group (Figures 4(e)–4(g)). In addition, the levels of 8-OHdG, 4-HNE, and MDA in the WT-VD14d group were significantly lower than their levels in the KO-VD14d group. Meanwhile, the activities of CAT, GSH-PX, and T-SOD in the WT-VD14d group were significantly higher than those in the KO-VD14d group. These results suggest that VD induced oxidative damage in the anterior vaginal walls of mice and that *Nrf2* knockout exacerbates this through the suppression of antioxidant activity.

### 3.5. Nrf2 Overexpression Alleviates the Mechanical Trauma-Induced Reduction of LPP and Increase in Apoptosis in Mice

**Figure 5:**
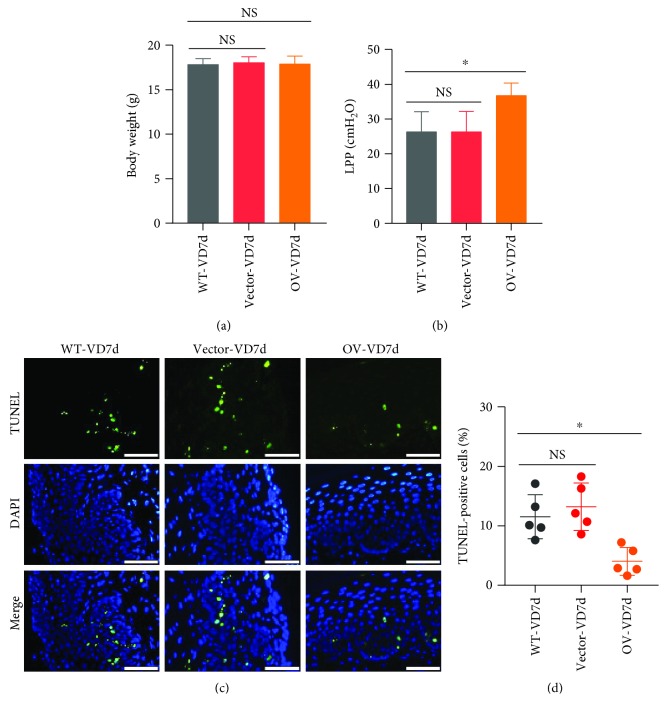
Effects of Nrf2 overexpression on mechanical trauma-induced LPP reduction and apoptosis. (a) Body weights of mice in three groups. (b) LPP values of mice in three groups. (c, d) TUNEL assay was used to detect the cell apoptosis in the anterior wall of mice in three groups. Green fluorescence is a positive signal for apoptosis. Scale bars: 50 *μ*m. ^∗^ *P* < 0.05; NS: no significant difference. Every experiment was repeated 3 times and representative figures are presented here. LPP: leak point pressure; VD: vaginal distension; WT-VD7d: mice in the control group and evaluated on day 7 after VD; Vector-VD7d: mice transfected with LV vector and evaluated on day 7 after VD; Nrf2-OV: mice transfected with LV-Nfe2l2 for *in vivo* Nrf2 overexpression and evaluated on day 7 after VD.

In order to validate the protective role of Nrf2 in mechanical trauma-induced SUI, we performed *in vivo* transfection of WT mice to establish a Nrf2-overexpressing mouse model. As shown in Figure 5(a), there were no significant differences in body weight among the three groups of mice. After VD, the LPP value in the Nrf2-overexpression group was significantly higher than the LPP value in the control group (Figure 5(b)). On the contrary, apoptosis decreased in the Nrf2-overexpression group compared with the control group after VD (Figures 5(c) and 5(d)). These results suggest that Nrf2 overexpression, to some extent, reversed mechanical trauma-induced reduction of LPP and increase in apoptosis.

### 3.6. Nrf2 Overexpression Alleviates Mechanical Trauma-Induced Oxidative Damage in the Anterior Vaginal Walls of Mice

**Figure 6:**
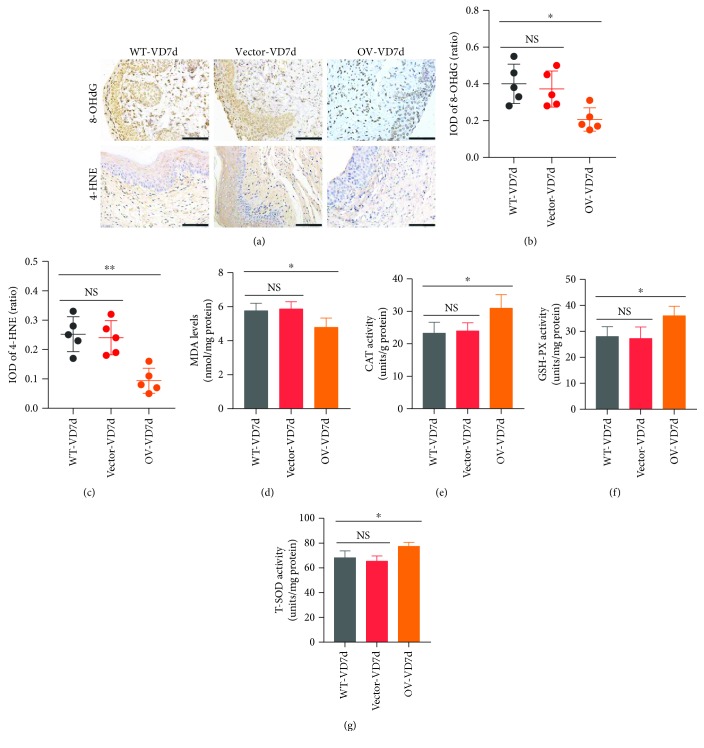
Effects of Nrf2 overexpression on mechanical trauma-induced alterations of oxidative damage and antioxidative capacity. (a–c) Immunohistochemistry was used to detect the levels of 8-OHdG and 4-HNE in the anterior wall of mice in three groups. Brown represents the positive signal. Scale bars: 100 *μ*m. (d) The MDA measurement kit was used to detect the levels of MDA in the anterior wall of mice in three groups. (e–g) CAT, GSH-PX, and T-SOD measurement kits were used to detect the activities of CAT, GSH-PX, and T-SOD, respectively, in the anterior wall of mice in three groups. ^∗^ *P* < 0.05; NS: no significant difference. Every experiment was repeated 3 times and representative figures are presented here. 8-OHdG: 8-oxo-2′-deoxyguanosine; 4-HNE: 4-hydroxynonenal; MDA: malondialdehyde; CAT: catalase; GSH-PX: glutathione peroxidase; T-SOD: total superoxide dismutase; VD: vaginal distension; WT-VD7d: mice in control group and evaluated on day 7 after VD; Vector-VD7d: mice transfected with LV vector and evaluated on day 7 after VD; Nrf2-OV: mice transfected with LV-Nfe2l2 for *in vivo* Nrf2 overexpression and evaluated on day 7 after VD.

As shown in Figures 6(a)–6(d), the levels of 8-OHdG, 4-HNE, and MDA in the Nrf2-overexpression group were significantly lower than those in the control group after VD. The activities of CAT, GSH-PX, and T-SOD were also significantly higher in the Nrf2-overexpression group than in the control group after VD (Figures 6(e)–6(g)). These results indicate that Nrf2 overexpression, to some extent, alleviated the mechanical trauma-induced oxidative damage and decrease in antioxidative capacity.

## 4. Discussion

Vaginal delivery is one of the most importantly recognized, independent risk factors for SUI, but the exact pathogenesis of vaginal delivery-related SUI is still not clear. Mechanical stretch and extrusion of pelvic tissues during vaginal delivery can injure pelvic connective tissues, muscles, and nerves, which are responsible for maintaining continence [22, 23]. Oxidative damage and apoptosis are reported to be involved in the pathological process of PFD [8, 9, 21]. Our previous research has shown that excessive mechanical stress induces oxidative damage and results in apoptosis in hUSLFs and L929 fibroblasts [8, 17, 21]. Nrf2 is an upstream transcription factor that modulates the activation of antioxidant cascades. Therefore, in this study, we explored the potential protective role of Nrf2 in mechanical trauma-induced SUI *in vivo*. The results showed that mechanical trauma can induce oxidative damage in the anterior vaginal walls of mice, thus promoting apoptosis and resulting in slackness of the pelvic supporting tissues. However, Nrf2 overexpression can alleviate mechanical injury-induced oxidative damage and apoptosis, to some extent, via the enhancement of antioxidative capacity.

VD is the most commonly used method for simulating SUI in rodent models, and urodynamic testing of LPP is the most commonly used method for the diagnosis and evaluation of SUI in rodent models [24–26]. In our previous study, we found that the LPP of mice markedly decreased on day 7 after VD and then began to recover from day 14 [20]. Based on this result, we evaluated the protective role of Nrf2 in the mechanical trauma-induced SUI model via LPP measurements on days 7 and 14 after VD. Our results showed that *Nrf2* knockout aggravated VD-induced LPP reduction and delayed the recovery of LPP in mice. Meanwhile, *in vivo* Nrf2 overexpression rescued VD-induced LPP reduction, to some extent. These results indicate that Nrf2 may have a protective effect on mechanical trauma-induced SUI.

To further understand the molecular mechanism of the protective role of Nrf2, apoptosis and oxidative damage were evaluated in the anterior vaginal walls of WT and *Nrf2*-knockout mice after VD. The results showed that *Nrf2* knockout exacerbated VD-induced apoptosis and activation of apoptosis signaling and delayed the decline in apoptosis rate and activation of apoptosis signaling in the anterior vaginal walls of mice. The increase in apoptosis in pelvic supporting tissues is strongly related with their slackness, their abnormal anatomical position, and the function of pelvic organs [9–11]. The bladder neck and urethra are attached to the anterior wall of the vagina, and the vaginal wall is connected to the levator ani muscle by the arcus tendineus fasciae pelvis [7]. According to the pelvic integral theory, vaginal delivery and chronic increase in abdominal pressure can induce mechanical injury to pelvic connective tissues and muscles, which can cause traumatic slackness and alteration in the normal anatomical position and function of these tissues, thus resulting in SUI [5, 6, 22]. Hence, increase of apoptosis in the anterior vaginal wall may contribute to the anatomic abnormality and dysfunction of the vesical neck and urethra. Kim et al. reported that the rate of apoptosis and the expression of cleaved caspases-3 and -9 were significantly higher in uterosacral ligaments of patients with stage III or IV POP compared to those with stage 0 or I POP [9]. Furthermore, Resplande et al. [27] confirmed that the apoptotic index increased significantly in the urethra of castrated/ballooning rats, with a predominance in the submucosa layer, and this was accompanied by a reduction in LPP compared with the control group. All of these results are consistent with our findings in this study. Therefore, the mechanical injury-induced increase in apoptosis in the mouse anterior vaginal wall may be one of the most important causes of mechanical trauma-induced SUI. In addition, VD increased the levels of 8-OHdG, 4-HNE, and MDA but decreased the activities of CAT, GSH-PX, and T-SOD in the anterior wall. *Nrf2* knockout significantly aggravated VD-induced upregulation of peroxidation products and the decrease in antioxidative capacity in the mouse anterior vaginal wall. Similarly, the reduction in peroxidation products and increase in antioxidative capacity were also delayed.

Based on the above results, we can deduce that an oxidant-antioxidant imbalance and the consequent oxidative damage in pelvic tissues may be involved in mechanical trauma-induced apoptosis and SUI. Furthermore, Nrf2 may have a protective role in these pathological changes. To further confirm this hypothesis, a mouse model with Nrf2 overexpression was established. Our data indicated that Nrf2 overexpression increased LPP and antioxidative activities in mice on day 7 after VD. On the contrary, Nrf2 overexpression decreased apoptosis and oxidative damage in the anterior vaginal wall of mice on day 7 after VD. Chen et al. reported that ginsenoside Rh2 showed a protective role against mechanical injury-induced SUI via upregulation of SOD3 and downregulation of nitric oxide synthase (iNOS) [25]. Similarly, our previous study indicated that punicalagin, an antioxidant, also has a protective role in VD-induced oxidative damage and LPP reduction [20].

In summary, the results of this study indicate that mechanical trauma simulated by VD can increase apoptosis in the anterior vaginal walls of mice via upregulation of oxidative damage. In addition, Nrf2 has a significant protective role in mechanical trauma-induced apoptosis in a mouse model of SUI, through the upregulation of antioxidative capacity and the consequent decrease in oxidative damage. Therefore, Nrf2 may be an important target for SUI prevention and treatment and antioxidative therapy may be a promising treatment for SUI. However, further research on the protective role of Nrf2 against mechanical trauma-induced muscular and nervous tissue injury in the SUI model is needed to further confirm its important role in the prevention and treatment of SUI.

## Data Availability

The data used to support the findings of this study are available from the corresponding author upon request.

## Supplementary Materials

Supplementary Materials Supplementary data include the details of the identification and validation of Nfe2l2^−/−^ mice (Figure S1); details of the validation of the *in vivo* transfection (Figure S2); and details of vaginal distention, suprapubic tube implantation, LPP measurement, TUNEL assay, western blot analysis, MDA assay, CAT assay, GSH-PX assay, T-SOD assay, and immunohistochemistry assay.

## References

[1] Lavelle E. S., Zyczynski H. M. (2016). Stress urinary incontinence: comparative efficacy trials. *Obstetrics and Gynecology Clinics of North America*.

[2] Syan R., Brucker B. M. (2016). Guideline of guidelines: urinary incontinence. *BJU International*.

[3] Barber M. D., Maher C. (2013). Epidemiology and outcome assessment of pelvic organ prolapse. *International Urogynecology Journal*.

[4] Zhu L., Lang J., Liu C., Han S., Huang J., Li X. (2009). The epidemiological study of women with urinary incontinence and risk factors for stress urinary incontinence in China. *Menopause*.

[5] DeLancey J. O. L. (2002). Fascial and muscular abnormalities in women with urethral hypermobility and anterior vaginal wall prolapse. *American Journal of Obstetrics and Gynecology*.

[6] DeLancey J. O. L. (1994). Structural support of the urethra as it relates to stress urinary incontinence: the hammock hypothesis. *American Journal of Obstetrics and Gynecology*.

[7] Parker-Autry C. Y., Burgio K. L., Richter H. E. (2012). Vitamin D status: a review with implications for the pelvic floor. *International Urogynecology Journal*.

[8] Hong S., Hong L., Li B. (2017). The role of GPX1 in the pathogenesis of female pelvic organ prolapse. *PLoS One*.

[9] Kim E. J., Chung N., Park S. H. (2013). Involvement of oxidative stress and mitochondrial apoptosis in the pathogenesis of pelvic organ prolapse. *The Journal of Urology*.

[10] Strasser H., Tiefenthaler M., Steinlechner M., Eder I., Bartsch G., Konwalinka G. (2000). Age dependent apoptosis and loss of rhabdosphincter cells. *Journal of Urology*.

[11] Takacs P., Gualtieri M., Nassiri M., Candiotti K., Medina C. A. (2008). Vaginal smooth muscle cell apoptosis is increased in women with pelvic organ prolapse. *International Urogynecology Journal and Pelvic Floor Dysfunction*.

[12] Chen Y. J., Jeng J. H., Chang H. H., Huang M. Y., Tsai F. F., Jane Yao C. C. (2013). Differential regulation of collagen, lysyl oxidase and MMP-2 in human periodontal ligament cells by low- and high-level mechanical stretching. *Journal of Periodontal Research*.

[13] Froese A. R., Shimbori C., Bellaye P. S. (2016). Stretch-induced activation of transforming growth factor-*β*
_1_ in pulmonary fibrosis. *American Journal of Respiratory and Critical Care Medicine*.

[14] Ghantous C. M., Kobeissy F. H., Soudani N. (2015). Mechanical stretch-induced vascular hypertrophy occurs through modulation of leptin synthesis-mediated ROS formation and GATA-4 nuclear translocation. *Frontiers in Pharmacology*.

[15] Tan J., Xu X., Tong Z. (2015). Decreased osteogenesis of adult mesenchymal stem cells by reactive oxygen species under cyclic stretch: a possible mechanism of age related osteoporosis. *Bone Research*.

[16] Tang J., Li B., Liu C. (2017). Mechanism of mechanical trauma-induced extracellular matrix remodeling of fibroblasts in association with Nrf2/ARE signaling suppression mediating TGF-*β*1/Smad3 signaling inhibition. *Oxidative Medicine and Cellular Longevity*.

[17] Li Q., Li B., Liu C., Wang L., Tang J., Hong L. (2018). Protective role of Nrf2 against mechanical-stretch-induced apoptosis in mouse fibroblasts: a potential therapeutic target of mechanical-trauma-induced stress urinary incontinence. *International Urogynecology Journal*.

[18] Morizono K., Xie Y., Ringpis G. E. (2005). Lentiviral vector retargeting to P-glycoprotein on metastatic melanoma through intravenous injection. *Nature Medicine*.

[19] Yu H., Zhao G., Li H., Liu X., Wang S. (2012). Candesartan antagonizes pressure overload-evoked cardiac remodeling through Smad7 gene-dependent MMP-9 suppression. *Gene*.

[20] Tang J., Liu C., Min J., Hu M., Li Y., Hong L. (2017). Potential therapeutic role of punicalagin against mechanical-trauma-induced stress urinary incontinence via upregulation of Nrf2 and TGF-*β*1 signaling: effect of punicalagin on mechanical trauma induced SUI. *International Urogynecology Journal*.

[21] Hong S., Hong L., Wu D. (2015). Oxidative damage to human parametrial ligament fibroblasts induced by mechanical stress. *Molecular Medicine Reports*.

[22] Dietz H. P. (2013). Pelvic floor trauma in childbirth. *The Australian & New Zealand Journal of Obstetrics & Gynaecology*.

[23] Gaski G., Frantz T., Steenburg S., Bell T., McKinley T. (2016). Large-magnitude pelvic and retroperitoneal tissue damage predicts organ failure. *Clinical Orthopaedics and Related Research*.

[24] Chen H. Y., Chen C. J., Lin Y. N., Chen Y. H., Chen W. C., Chen C. M. (2013). Proteomic analysis related to stress urinary incontinence following vaginal trauma in female mice. *European Journal of Obstetrics, Gynecology, and Reproductive Biology*.

[25] Chen Y. H., Lin Y. N., Chen W. C., Hsieh W. T., Chen H. Y. (2014). Treatment of stress urinary incontinence by ginsenoside Rh2. *The American Journal of Chinese Medicine*.

[26] Oliveira D. A., Parente M. P. L., Calvo B., Mascarenhas T., Natal Jorge R. M. (2016). Numerical simulation of the damage evolution in the pelvic floor muscles during childbirth. *Journal of Biomechanics*.

[27] Resplande J., Gholami S. S., Graziottin T. M. (2002). Long-term effect of ovariectomy and simulated birth trauma on the lower urinary tract of female rats. *The Journal of Urology*.

