# Supplementary material for: Protective Role of Nuclear Factor Erythroid-2-Related Factor 2 against Mechanical Trauma-Induced Apoptosis in a Vaginal Distension-Induced Stress Urinary Incontinence Mouse Model
Source: Oxid Med Cell Longev. 2019 Mar 6;2019:2039856. doi: 10.1155/2019/2039856 (PMC6431382; doi:10.1155/2019/2039856)
Supplement: Supplementary Materials — Supplementary data include the details of the identification and validation of Nfe2l2−/− mice (Figure S1); details of the validation of the in vivo transfection (Figure S2); and details of vaginal distention, suprapubic tube implantation, LPP measurement, TUNEL assay, western blot analysis, MDA assay, CAT assay, GSH-PX assay, T-SOD assay, and immunohistochemistry assay. [file 2039856.f1.pdf]

Supplementary Materials

S1. Materials and methods

Vaginal distention Mice in the VD groups underwent vaginal distention after being anesthetized with urethane (1g/kg, i.p.). After lubrication with paraffin oil, a modified 6-Fr. Foley catheter was inserted into vagina and secured to the vaginal introitus with a 5/0 silk suture. Then, 0.3 ml distilled water was infused into the balloon to distend the vagina. Each balloon's diameter was measured before VD using a Vernier caliper. After 1 h, the balloon was deflated and removed, and the mouse permitted to wake spontaneously. The NC group did not undergo VD and sham groups just being inserted with a modified 6-Fr. Foley catheter into the vagina and secured with a 5/0 silk suture without distilled water infusion.

Suprapubic Tube Implantation and LPP Measurement One day before LPP measurement, an epidural catheter was implanted in the bladder under urethane (1 g/kg, i.p.) anesthesia. On the day of LPP measurement, mice were again anesthetized with urethane (1 g/kg, i.p.), the bladder catheter was connected to both a micro syringe pump and a pressure transducer of urinary dynamics detector (Nidoc970C, Weixin Medical of China) through a T-branch pipe. Pressure and force transducer signals were amplified and digitized for computer data collection. The bladder was then filled with room-temperature saline at 1 ml/h through the bladder catheter. When half the bladder capacity was reached, gentle pressure with one finger was applied to the mouse's abdomen. Pressure was gently increased until urine leaked, at which time the externally applied pressure was rapidly removed. Peak bladder pressure was used as the LPP. Voids could be easily distinguished from leaks. If a mouse voided, the bladder was refilled and the process was repeated. At least five LPPs were obtained on each animal and the mean calculated.

TUNEL assay for the detection of apoptosis Cell apoptosis of anterior vaginal wall of mice were detected using the ApopTag Plus Fluorescein In Situ Apoptosis Detection Kit S7111 (Chemicon, Temecula, California) according to manufacturer's instructions. All specimens were embedded in paraffin and cut into 4 μm thick and fixed onto glass slides. There were 9 steps in this experiment. Step 1: the specimens were washed in xylene for three times, 5 min each, followed by two washes in absolute ethanol for 5 min each, one wash in 95% ethanol for 3 min, one in 70% ethanol for 3 min, and a final wash in PBS for 5 min; Step 2: the specimens were treated with freshly diluted Proteinase K (20 μg/mL) for 15 min at room temperature in a Coplin jar, then washed in two changes of PBS for 2 min each wash; Step 3: equilibration buffer (75 uL/5 cm^2) was applied on the specimen and followed by 10 s' incubation at room temperature; Step 4: excess liquid was tapped off gently, then working strength TdT enzyme (55 μL/5 cm^2) was immediately pipette onto the section followed by 1 h's incubation in a humidified chamber at 37℃; Step 5: the specimens were placed in a Coplin jar containing working strength stop/wash buffer and followed by 15 s of agitation and 10 min of incubation at room temperature. During this incubation, the vial of anti-digoxigenin conjugate was brought to room temperature while avoiding any exposure to light; Step 6: the specimens were washed in PBS (pH 7.4) for three times, 1 min each. Then, excess liquid

was tapped off gently and carefully and working strength anti-digoxigenin conjugate was applied to the slide with 65 μL/5 cm^2 of surface covered, and followed by 30 min of incubation in a humidified chamber at room temperature away from the light; Step 7: the specimens were washed with four changes of PBS (pH 7.4) in a Coplin jar for 2 min each wash at room temperature; Step 8: a mounting medium containing 0.5 μg/mL of DAPI was applied and mounted with a glass cover slip; Step 9: specimens were viewed and imaged by fluorescence microscopy using filter for FITC (ex. 490 nm and em. 520 nm) and DAPI. The images were analyzed using Image-Pro Plus version 6.0 software (Media Cybernetics, Inc., Rockville, MD, USA) and the mean percentage of apoptosis positive cells in 200X microscopic field of every group were compared.

Western blot Details of the information and dilutions of antibodies are shown below：Bcl2: Abcam Inc., Cambridge, MA, USA (ab196495, 1:1,000); Bax: Abcam Inc., Cambridge, MA, USA (ab32503, 1:1,000); Caspase-3: Cell Signaling Technology, Inc., Danvers, MA, USA (9664, 1:1,000); Caspase-9: Cell Signaling Technology, Inc., Danvers, MA, USA (9508, 1:1,000); β-actin: Abcam Inc., Cambridge, MA, USA (ab8226, 1:2,000); Nrf2: Proteintech Inc., Wuhan, Hubei, P.R.C (16396-1-AP, 1:500); GAPDH: Abcam Inc., Cambridge, MA, USA (ab8245, 1:2,000).

MDA, CAT, GSH-PX and T-SOD measurement assay After total protein extraction from L929 cells and vaginal walls using RIPA buffer containing phenylmethylsulfonyl fluoride (PMSF). A BCA assay kit was used according to the manufacturer's instructions to detect the protein concentrations. Then, the cell extract was collected for measurements of human catalase activity by a catalase analysis kit following the manufacturer's instructions. Briefly, samples were treated with excess hydrogen peroxide for decomposition by catalase for an exact time, and the remaining hydrogen peroxide coupled with a substrate was treated with peroxidase to generate a red product, N-4-antipyryl-3-chloro-5-sulfonate-p-benzoquinonemonoimine, which absorbs maximally at 520 nm. Catalase activity was thus determined by measuring the decomposition of hydrogen peroxide spectrophotometrically. For lipid peroxidation assay, we used a malondialdehyde (MDA) analysis kit to quantify the generation of MDA according to the manufacturer's protocol. In brief, cells were harvested by trypsinization and cellular extracts were prepared by sonication in ice-cold buffer (50 mM Tris-HCl, pH 7.5, 5 mM EDTA, and 1 mM DTT). After sonication, lysed cells were centrifuged at 10,000×g for 20 min to remove debris. The supernatant was subjected to the measurement of MDA levels and the protein contents. MDA levels were then normalized to milligram protein. We used the same procedure to lyse the cells and determine the protein contents in the following assays unless otherwise indicated. For GSH-PX measurement, 200 μL specimen was added into 200 μL GSH solution (1 mmol/L), incubated in 37°C for 5 min, then add 100 μL of solution A and incubated in 37°C for 5 min, the next, add 2 mL of solution B and mixed. Then, centrifuge in 3500 rpm for 10 min and transfer 1 mL of supernate for chromogenic reaction, a control group was set in this step. The next step, 1 mL of solution C, 250 μL of solution D and 50 μL of solution E were added into1 mL of supernate one after another and mixed, then, let stand for another 15 min. The last, OD values in 412 nm were measured. A blank control, standard control and negative control were set respectively. The activity of GSH-PX was normalized to milligram protein. For T-SOD assay, 1 mL of solution A, B, C and D was

added into the specimen successively, and incubated in 37℃ for 40 min, then, 2 mL of chromogenic agent was added and let stand for 10 min. Last, the OD values were measured in 550 nm. The activity of T-SOD was normalized to milligram protein.

Immunohistochemistry Ultra-Sensitive SP kit-9710 (Maixin Biotech Co., Ltd., Fuzhou, China) was used for immunohistochemistry. The slides were heated for 30 min at 60°C, de-paraffinized and rehydrated in a graded alcohol series. Antigen retrieval was performed through boiling in citrate (pH <6) or EDTA buffer (pH >9), according to the product datasheets. This was followed by incubation with 3% H_2O_2 for 10 min at room temperature to inactivate endogenous peroxidase, and blocking with 5% goat serum for 15 min, and incubating with primary antibodies overnight at 4°C. Following incubation with streptavidin peroxidase for 10 min at room temperature, secondary antibodies were added. Finally, immune reaction was visualized using the DAB-0031 kit (Maixin Biotech Co., Ltd.). The specimens were washed with phosphate-buffered saline (PBS) after each step in the protocol. The primary antibody was replaced with PBS for the negative controls. The antibodies used were as follows: 8-OHdG: ab62623, 1:500 (Abcam Inc., Cambridge, MA, USA); 4-HNE: ab46465, 1:100 (Abcam Inc., Cambridge, MA, USA); Immunoactivity was quantified with the IOD, integrated optical density (IOD) value and the mean density was captured using Image-Pro Plus version 6.0 software (Media Cybernetics, Inc., Rockville, MD, USA); mean density = IOD/area.

S2. Figures

Figure S1:

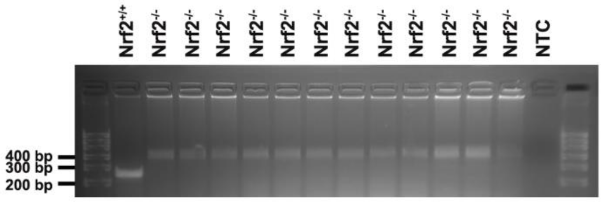

Figure S1. Validation of Nrf2 knockout mice (Nfe2l2-/-). Mice were validated via standard PCR according to the instruction of Jackson Laboratory (https://www2.jax.org/protocolsdb/f?p=116:5:0::NO:5:P5_MASTER_PROTOCOL_ID,P5_JRS_CODE:7474,017009) ; Mutant = ~400 bp, Heterozygote = ~400 bp and 262 bp, Wild type = 262 bp; (NTC: PCR reaction mixture control without DNA template)

Figure S2:

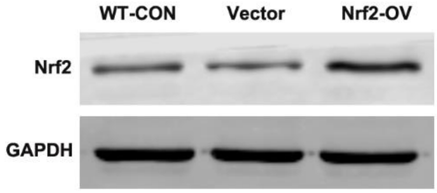

Figure S2. Validation of *in vivo* Nrf2 over-expressing in wild-type C57BL/6J mice. Western blot was

used to validated the effect of *in vivo* Nrf2 over-expressing, the primary antibody of Nrf2 was purchased from Proteintech (16396-1-AP). WT-CON: mice in control group; Vector: mice transfected with Lv-vector; Nrf2-OV: mice transfected with Lv-Nfe2l2 for *in vivo* Nrf2 over-expressing.

S3. Data Availability Statement：

All data used to support the findings of this study are available from the corresponding author upon request.
